# Supplementary material for: Clostridioides difficile Flagellin Activates the Intracellular NLRC4 Inflammasome
Source: Int J Mol Sci. 2022 Oct 15;23(20):12366. doi: 10.3390/ijms232012366 (PMC9604438; doi:10.3390/ijms232012366)
Supplement: Supplementary file 1 [file ijms-23-12366-s001.zip › ijms-1960066-supplementary.pdf]

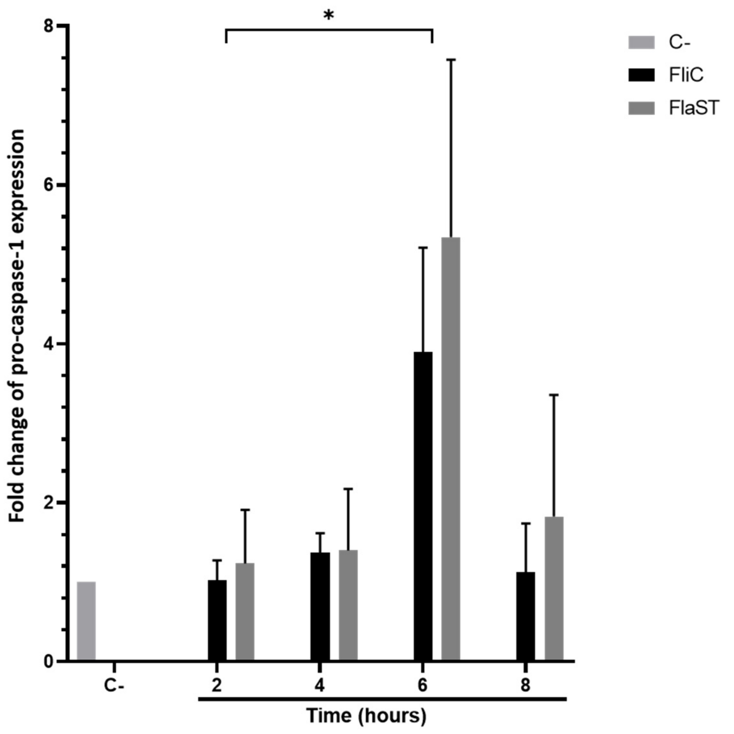

Figure S1. *C. difficile* flagellin induces pro-caspase-1 gene expression. Caco-2/TC7 cells treated by FliC (6 µg/mL), and FlaST (2 µg/mL) as a positive control. After 8 h, cell-free culture supernatants were collected, and pro-caspase-1 gene was quantified. Error bars indicate the standard error of the mean of three independent experiments. Results represent the mean ($n = 3$) ± standard deviations for each condition. * Statistically significant differences ($p < 0.05$).

*L. pneumophila**	A	E	M	A	S	L	T	K	N	Q	I	L	Q	Q	A	G	T	A	M	L	A	Q	A	N	S	L	P	Q	S	V	L	S	L	L	G	R
S. Typhimurium*	T	E	V	S	N	M	S	R	A	Q	I	L	Q	Q	A	G	T	S	V	L	A	Q	A	N	Q	V	P	Q	N	V	L	S	L	L	R	.
*Y. enterocolitica**	T	E	V	S	N	M	S	R	A	N	I	L	Q	Q	A	G	T	S	V	L	A	Q	A	N	Q	V	P	Q	T	V	L	S	L	L	R	.
*EPEC**	T	E	V	S	N	M	S	K	A	Q	I	L	Q	Q	A	G	N	S	V	L	A	K	A	N	Q	V	P	Q	Q	V	L	S	L	L	Q	G
*EHEC**	T	E	V	S	N	M	S	K	A	Q	I	L	Q	Q	A	G	N	S	V	L	A	K	A	N	Q	V	P	Q	Q	V	L	S	L	L	Q	G
*P. luminescens**	T	E	V	S	N	M	S	R	G	Q	I	L	Q	Q	A	G	T	A	V	L	A	Q	A	N	Q	V	P	Q	N	V	M	S	L	L	R	.
*B. thailandensis**	Q	E	T	A	N	L	S	R	A	Q	V	L	Q	Q	A	G	I	S	V	L	A	Q	A	N	S	L	P	Q	Q	V	L	K	L	L	Q	.
*P. aeroginosa**	A	E	T	A	A	L	S	K	N	Q	V	L	Q	Q	A	G	T	A	I	L	A	Q	A	N	Q	L	P	Q	A	V	L	S	L	L	R	.
*C. difficile***	E	M	V	A	N	L	S	K	M	N	I	L	V	Q	A	S	Q	S	M	L	A	Q	A	N	Q	Q	P	Q	G	V	L	Q	L	L	G	.
												★		★	★		★		★		★							★	★	★	★	★	★	★	★	

Figure S2. Flagellins interact with inflammasomes via a conserved region: Sequence alignment of the C-terminal sides of different bacterial flagellins. Flagellin residues interacting with NAIP5 are indicated with asterisks. Conserved residues are in red. Amino acids localizations are 461-495 (*) or 241-290 (**). According to Yang et al. [36].
